# The role of co-production in Learning Health Systems

**DOI:** 10.1093/intqhc/mzab072

**Published:** 2021-11-29

**Authors:** Andreas Gremyr, Boel Andersson Gäre, Johan Thor, Glyn Elwyn, Paul Batalden, Ann-Christine Andersson

**Affiliations:** Department of Schizophrenia Spectrum Disorders, Sahlgrenska University Hospital, Sahlgrenska Universitetssjukhuset Psykiatri Psykos, Göteborgsvägen 31, Mölndal, Västragötalandsregionen 431 80, Sweden; Jönköping Academy for Improvement of Health and Welfare, School of Health and Welfare, Jönköping University, Barnarpsgatan 39, Jönköping, Jönköpings län 55111, Sweden; Jönköping Academy for Improvement of Health and Welfare, School of Health and Welfare, Jönköping University, Barnarpsgatan 39, Jönköping, Jönköpings län 55111, Sweden; Geisel School of Medicine at Dartmouth, The Dartmouth Institute for Health Policy and Clinical Practice, Williamson Translational Research Building, Level 5, 1 Medical Center Drive, Lebanon, NH 03756, USA; Geisel School of Medicine at Dartmouth, The Dartmouth Institute for Health Policy and Clinical Practice, Williamson Translational Research Building, Level 5, 1 Medical Center Drive, Lebanon, NH 03756, USA; Jönköping Academy for Improvement of Health and Welfare, School of Health and Welfare, Jönköping University, Barnarpsgatan 39, Jönköping, Jönköpings län 55111, Sweden; Geisel School of Medicine at Dartmouth, The Dartmouth Institute for Health Policy and Clinical Practice, Williamson Translational Research Building, Level 5, 1 Medical Center Drive, Lebanon, NH 03756, USA; Jönköping Academy for Improvement of Health and Welfare, School of Health and Welfare, Jönköping University, Barnarpsgatan 39, Jönköping, Jönköpings län 55111, Sweden; Department of Care Science, Malmö University, Nordenskiöldsgatan 1, Malmö, Skåne 211 19, Sweden

**Keywords:** Learning Health System, patient-centred care, health quality improvement, health service research, co-production

## Abstract

**Background:**

Co-production of health is defined as ‘the interdependent work of users and professionals who are creating, designing, producing, delivering, assessing, and evaluating the relationships and actions that contribute to the health of individuals and populations’. It can assume many forms and include multiple stakeholders in pursuit of continuous improvement, as in Learning Health Systems (LHSs). There is increasing interest in how the LHS concept allows integration of different knowledge domains to support and achieve better health. Even if definitions of LHSs include engaging users and their family as active participants in aspects of enabling better health for individuals and populations, LHS descriptions emphasize technological solutions, such as the use of information systems. Fewer LHS texts address how interpersonal interactions contribute to the design and improvement of healthcare services.

**Objective:**

We examined the literature on LHS to clarify the role and contributions of co-production in LHS conceptualizations and applications.

**Method:**

First, we undertook a scoping review of LHS conceptualizations. Second, we compared those conceptualizations to the characteristics of LHSs first described by the US Institute of Medicine. Third, we examined the LHS conceptualizations to assess how they bring four types of value co-creation in public services into play: co-production, co-design, co-construction and co-innovation. These were used to describe core ideas, as principles, to guide development.

**Result:**

Among 17 identified LHS conceptualizations, 3 qualified as most comprehensive regarding fidelity to LHS characteristics and their use in multiple settings: (i) the Cincinnati Collaborative LHS Model, (ii) the Dartmouth Coproduction LHS Model and (iii) the Michigan Learning Cycle Model. These conceptualizations exhibit all four types of value co-creation, provide examples of how LHSs can harness co-production and are used to identify principles that can enhance value co-creation: (i) use a shared aim, (ii) navigate towards improved outcomes, (iii) tailor feedback with and for users, (iv) distribute leadership, (v) facilitate interactions, (vi) co-design services and (vii) support self-organization.

**Conclusions:**

The LHS conceptualizations have common features and harness co-production to generate value for individual patients as well as for health systems. They facilitate learning and improvement by integrating supportive technologies into the sociotechnical systems that make up healthcare. Further research on LHS applications in real-world complex settings is needed to unpack how LHSs are grown through coproduction and other types of value co-creation.

## Background

With the great challenges that healthcare service systems face, there has been an increased emphasis on co-production of health. Batalden [1] proposes that health is not a product generated by professionals in the healthcare system but a service co-produced with users, e.g. patients. Co-production of health is defined as ‘the interdependent work of users and professionals who are creating, designing, producing, delivering, assessing, and evaluating the relationships and actions that contribute to the health of individuals and populations’ [1]. It carries with it a new focus on the logic of making a service [2]. There are many applications of the original idea of co-production [3–5]. Elwyn *et al*. connect co-production to the voice of the patient, to practice improvement and organizational design of a Learning Health System (LHS) [6].

The idea of an LHS was introduced in 2007 by the US Institute of Medicine (IoM, now the National Academies of Medicine) to better serve the needs of patients and those who support them. It suggested that by combining the strengths of many different knowledge domains, continuous improvements could be enabled. An LHS is defined as a system ‘in which science, informatics, incentives, and culture are aligned for continuous improvement and innovation, with best practices seamlessly embedded in the care process, patients and families active participants in all elements, and new knowledge captured as an integral by-product of the care experience’ [7]. Even though to some extent LHS-like healthcare systems have been developed both before and after IoM introduced the idea, but framed differently, e.g. as learning communities [8], the LHS idea provided an umbrella term and some sought for characteristics [7]. Since the time of the original report, the idea of co-production and its attendant service-making logic offers an unexplored and potentially helpful contribution to an idea of an LHS.

Reviewing the recent LHS literature, Platt *et al*. [9] found a focus on technical solutions. Fewer studies addressed the role of interpersonal interactions in LHSs viewed as complex ecosystems promoting health. We, therefore, examined the literature on LHS to clarify the role and contributions of co-production in a selection of illustrative examples of comprehensive LHS conceptualizations and related applications.

## Methods

We selected, reviewed and assessed examples of LHS conceptualizations regarding co-production in three steps:

First, we undertook a scoping review, based on Arksey and O’Malley’s study [10], to search and select LHS literature. A university librarian helped develop a comprehensive search strategy, including the choice of databases, MeSH terms and keywords, and performed searches in four databases (Cochrane, PubMed, PsychInfo and IEEE Xplore), using two queries: ‘learning health’ and ‘learning healthcare’. English-language articles published from 1 January 2007 to 9 March 2020 were screened for relevance by reading titles and abstracts. Full-text articles describing conceptualizations and applications of LHSs were reviewed independently by two authors.

Second, to identify comprehensive LHS conceptualizations, the identified LHS conceptualizations were assessed, independently by two authors, regarding their origin, content, articles showing utility/spread and fidelity to the LHS characteristics laid down by the IoM [7] (See box 1).

Box 1 **Characteristics of a continuously learning healthcare system [7]**

Science and informatics

- Real-time access to knowledge—A learning healthcare system continuously and reliably captures, curates and delivers the best available evidence to guide, support, tailor and improve clinical decision-making and care safety and quality.
- Digital capture of the care experience—A learning healthcare system captures the care experience on digital platforms for real-time generation and application of knowledge for care improvement.

Patient–clinician partnerships

- Engaged, empowered patients—A learning healthcare system is anchored on patient needs and perspectives and promotes the inclusion of patients, families and other caregivers as vital members of the continuously learning care team.

Incentives

- Incentives aligned for value—A learning healthcare system has incentives actively aligned to encourage continuous improvement, identify and reduce waste, and reward high-value care.
- Full transparency—A learning healthcare system systematically monitors the safety, quality, processes, prices, costs and outcomes of care and makes information available for care improvement and informed choices and decision-making by clinicians, patients and their families.

Continuous learning culture

- Leadership-instilled culture of learning—A learning healthcare system is stewarded by leadership committed to a culture of teamwork, collaboration and adaptability in support of continuous learning as a core aim.
- Supportive system competencies—A learning healthcare system constantly refines complex care operations and processes through ongoing team training and skill building, systems analysis and information development, and creation of the feedback loops for continuous learning and system improvement.

Third, three conceptualizations, with high fidelity to the IoM’s LHS characteristics and evidence of application in multiple settings to develop LHSs, were selected as illustrative examples. These were reviewed regarding co-production, drawing on Osborne *et al*.’s [3] recently described typology of co-production that can lead to improved services and value co-creation: coproduction, co-design, co-construction and co-innovation. We adapted their definitions to fit a healthcare service perspective specifically, as they address public services in general. Specifically co-construction and co-innovation were interpreted as including actors in addition to service users and professionals, e.g. next of kin (See Table 1).

**Table 1:** Osborne *et al*.’s [3] descriptions of four types of value co-creation and our adaptations for the healthcare context

	From Osborne *et al*. [3].	Adapted definitions for healthcare use
Co-production	The user co-produces the service experience and outcomes (public value) with public service staff	The joint activity of a service user and professional(s) in supporting and generating better health
Co-design	Improving the performance of existing public services by actively involving the service user in their design, evaluation and improvement	Improving the performance of existing health services by actively involving the service user in their design, evaluation and improvement
Co-construction	The co-creation of value by the individual well-being created through Type I (co-production) or Type II (co-design) activities, such as the well-being created for individuals as a result of helping them resolve the impact of a disability upon their life	The community building of service users, professionals and other stakeholders in supporting identity-building and sharing of resources to promote health
Co-innovation	The co-creation of social capital in an individual and/or community through co-production that co-creates capacity to resolve problems in the future	The co-creation of social capital in an individual and/or community through co-production that co-creates capacity to resolve problems in the future, creating new ways for the individuals and/or community to promote and support health

We then used the adapted four types of co-production to identify features of LHSs that can serve as principles guiding development of LHSs in conditions of complexity, inspired by Braun and Clarke [11].

## Results

The initial search yielded 839 unique publications, 24 of which contained 17 different conceptualizations (Chart in appendix). These 17 were assessed in relation to fidelity to the IoM LHS characteristics and the utilization of the conceptualizations in practice. Three LHS conceptualizations stood out as most comprehensive: (i) the Cincinnati Collaborative LHS Model, (ii) the Dartmouth Coproduction LHS Model and (iii) the Michigan Learning Cycle Model. They are presented below regarding their origin, content and utility. They were further analysed in relation to the four types of healthcare value co-creation (Table 1) to identify the role of co-production in comprehensive LHSs (Table 2).

**Table 2:** Concepts, activities and functions identified in the three most comprehensive LHS conceptualizations and how they relate to four types of value co-creation, inspired by Osborne *et al*.’s framework [3]

Co-creation of value	Concepts, activities and functions in the LHS models
I. Co-production The joint activity of a service user and professional(s) in supporting and generating better health.	Focus on what matters for the patient, guided by outcome measures, preferably patient-reported [6, 16, 21, 22, 29, 37, 38] Information and interfaces tailored to user needs: supporting joint planning, evaluation of progress, continuous learning and improvement [21, 22, 29, 30, 39, 40] with individualized information on prognosis, risk and treatment options, based on the experience of individuals with similar characteristics (real-time, and real world, evidence, i.e. ‘personalized medicine’) [29, 30, 41] SDM or other structured methods to support patient coproduction [6] including family and friends when appropriate [22] supporting patients to voice concerns and questions [22, 27]
II. Co-design Improving the performance of existing health services by actively involving the service user in their design, evaluation and improvement.	Use of user-/human-centred design to adapt services and interfaces [14, 27, 29, 42, 43] Continuous improvement using collaborative improvement networks and quality improvement methodologies [16, 21, 22] Use of feedback to support continuous learning and improvement in quality improvement projects, care teams and management [16, 21, 22, 29, 30, 34, 44, 45]
III. Co-construction The community building of service users, professionals and other stakeholders in supporting identity-building and sharing of resources to promote health.	Building community, across stakeholders [16, 17, 22, 29] Sharing resources, experiences and know-how [13, 16, 29, 39] Supporting interactions, using platforms [13, 14, 16]
IV. Co-innovation The co-creation of social capital in an individual and/or community through co-production that co-creates capacity to resolve problems in the future, creating new ways for the individuals and/or community to promote and support health.	Aligning efforts to provide better health and care for patients, support for clinicians, overview for managers and more effective research, through a shared aim [16, 22, 29, 30, 39] Transforming the health system by using commons-based peer production, coordinated into large, meaningful projects [22, 29, 39, 42] Supporting self-organization, e.g. to undertake improvement efforts [13, 16, 22, 29, 46]

### The Cincinnati Collaborative LHS Model [12, 13]

The Cincinnati Collaborative LHS Model [12, 13] builds on the ideas of Learning Networks, which in turn draw on the Chronic Care Model [14, 15] and use actor-oriented network architecture [13, 16], initially conceptualized as Collaborative Chronic Care Networks to address complex chronic illnesses [17]. The actor-oriented network organizational approach has three core components [16]: (i) a common goal to align participants, (ii) multi-actor collaboration facilitated through standards, processes, policies and infrastructure and (iii) a commons where information, knowledge, resources and know-how are created and shared to achieve the common goal. The most prominent example, the ImproveCareNow (ICN) network—originated at Cincinnati Children’s Hospital in the USA—aims to improve health outcomes for children and adolescents with Inflammatory Bowel Disease (IBD) and has co-evolved along with this LHS model. The network continues to spread across the USA and around the globe. ICN participants share goals, standards and resources and their continuous use of measurements demonstrate success, including continually improved clinical outcomes. The ICN has served as a prototype for a national paediatric LHS, the PEDSnet [18, 19]. No images of the Cincinnati Collaborative LHS Model are presented in the papers we reviewed. Nevertheless, the CLHS is the most elaborated model in terms of the number of supporting publications; of which some are showing improved clinical outcomes [16, 20].

### The Dartmouth Coproduction LHS Model [21]

**Figure 1:**
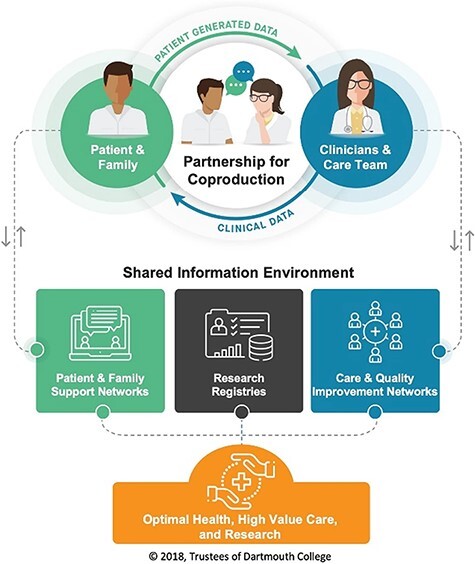
The Coproduction LHS model [21] (used with permission).

The Coproduction LHS Model highlights feed-forward systems for health information, the necessity of involving patients in setting health goals, making shared decisions, as well as measuring and evaluating outcomes to promote health, healthcare value and science. Researchers at the Dartmouth Institute for Health Policy and Clinical Practice in the USA have been central in developing the model [22], collaborating both locally and internationally, e.g. with the Dartmouth Spine Center and the Swedish Rheumatology Quality Register (SRQ). SRQ is one example where the model is associated with improved clinical outcomes [22]. The model has evolved in parallel with, and been influenced by, Clinical Microsystem theory and practice [23], approaches to shared decision-making (SDM) [24] and the Clinical Value Compass framework [25]. The model has been visualized in different versions with the latest shown in Figure 1 [21]. It emphasizes the coproduction of health in the shared space between patient (and family) and clinicians (and care team). This is accompanied by an enriched information environment intended to strengthen quality improvement collaboratives for clinical programs, facilitated support networks for patients and families, patient-centred registries and research efforts. Patient-reported outcome measures and other quality and value metrics support SDM and coproduction of health more broadly. The Coproduction LHS Model has been applied and studied for diverse, often complex, conditions including IBD [26], Cystic Fibrosis [21, 27] and Multiple Sclerosis [28], in palliative care [29] and is currently being adapted to support treatment of rheumatology in Europe and HIV in Africa.

### The Michigan Learning Cycle LHS model (LC-model) [30]

**Figure 2:**
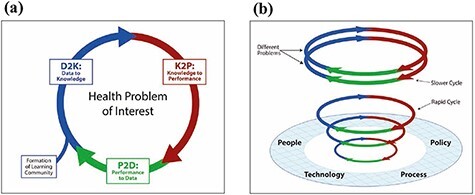
(a) The learning cycle. (b) The learning cycles platform [30] (used with permission).

The Michigan Learning Cycle LHS Model emphasizes continuous learning and improvement through learning cycles turning performance to data, data to knowledge and knowledge to performance (Figure 2a). Being the most recent of the three LHS models, it represents the highest level of abstraction and has primarily an academic origin, from the University of Michigan [30]. It has not specifically been formulated in tandem with any practical LHS initiative like the other two models. It suggests that learning cycles are to be applied to all kinds of challenges and problems in health care. By measuring and analysing data to yield new knowledge and understanding, which prompt action, application of the LC-Model aims to generate a virtuous cycle of improvement. Feedback loops are supported by a platform of people, policy, technology and process (Figure 2b). Rather than viewing the model as mechanistic, Friedman *et al*. propose that LC-Model LHSs have organic, or even fractal, properties, in that LHSs can start as small, parallel initiatives that grow independently and gradually connect, provided that they use the same standards. The LC-model has been used to explain how LHSs work [31] and how LHSs of the future could be developed, and it has served as a theory of change in developing LHS applications internationally, both in local/regional [32, 33] and national applications [34–36].

### Towards the co-creation of value in LHSs

Table 2 relates the conceptualizations and applications of the three most comprehensive LHSs to the four types of value co-creation based on our adaptation of Osborne *etal*.’s framework [3].

Inspired by thematic analysis [11], we developed the following guides to LHS development enhanced by co-creation:

1. Use a common attractor, a shared aim for stakeholders, to help build community, to guide efforts and to evaluate progress.
2. Navigate towards improved outcomes both for individual patients and for the system overall.
3. Use feedback tailored to specific users to inform treatments, quality improvement efforts, governance and research.
4. Enable distributed leadership so actors—patients, professionals or others—have a say in shaping decisions.
5. Facilitate interactions between individuals and groups to enable creative solutions and continuous improvement.
6. Apply co-design of services and user interfaces to make it easier for individuals and groups to engage in, and build, a culture of sharing, transparency and learning.
7. Support self-organization to create and share resources to transform the health system.

## Discussion

### General findings

This review of the LHS literature highlights three conceptualizations of LHSs, with high fidelity to the original LHS characteristics, that have also been applied in practice to develop LHSs. They employ many ways for users and professionals to generate and support better health and value through interactions, supported by other competencies, structures, technologies and ways to organize, when fully integrated by those users and professionals, even in conditions of complexity. They demonstrate the potential of the fundamental ‘role of co-production in LHSs’. The practical implications of the identified principles are further elaborated below.

### Practical implications

The three comprehensive LHS conceptualizations provide guidance beyond a single blueprint or set of guidelines, by exhibiting principles for usefulness in practice. The principles can clarify practical implications when developing LHSs: (i) use a common attractor, a shared aim for stakeholders, to help build community, to guide efforts and to evaluate progress. There are challenges in establishing a truly shared aim, which is not restricted by organizational boundaries but instead includes all relevant stakeholders including patients, family and friends, health and social care professionals, managers, and researchers. (ii) Navigate towards improved outcomes both for individual patients and for the system overall, which is essential since no guidelines can account for all aspects that can affect performance and outcomes in complex conditions. Monitoring outcomes that matter to the patient can support navigation towards improved services over all, as well as for the patient and their next of kin. (iii) Use feedback tailored to specific users to inform treatments, quality improvement efforts, governance and research. Feedback is useful when it provides new knowledge and prompts action when needed. Therefore, feedback needs to look differently for different users, depending on the purpose, whether it is, for instance, to support patients and healthcare professionals in co-designing treatments or out-patient unit managers in making priorities. (iv) Enable distributed leadership where actors—patients, professionals or others—have a say in shaping decisions. Letting the actors most closely involved with, and holding most of the relevant information, make decisions may challenge hierarchies and expert roles but has potential to enhance the co-creation of value. (v) Facilitate interactions between individuals and groups to enable creative solutions and continuous improvement. Helping people with similar interests to connect and communicate can bolster engagement. Communication between stakeholders, organizations and teams can yield better ideas on how to continuously improve care. (vi) Apply co-design of services and user interfaces to make it easier for individuals and groups to engage in, and build, a culture of sharing, transparency and learning. In a sociotechnical system, developing new ways of working, e.g. using new technologies to support care, is dependent on the users and their experience. Involving users is key. (vii) Support self-organization through platforms, with social media-like properties, to create and share resources to transform the health system. By Providing ways not only to communicate but to create, share and jointly test new ideas can help tap into the engagement of many, as actor-based networks, and support innovation.

### Limitations and challenges

This is not a complete description of what is important when developing LHSs. Instead, we focused here on the social part of the sociotechnical aspect of LHSs, i.e. how stakeholders’ co-production of treatments, quality improvement and research can enable better care. Osborne *et al*. [3] note that professionals play equally important roles as service users in the co-creation of value (without elaborating how) and do not explicitly describe how others than users and providers can contribute. The three comprehensive LHS conceptualization ways of enabling joint value creation are not restricted to the role of users or professionals but expand coproduction to involve ‘all’ stakeholders, e.g. family and friends, clinicians, managers and researchers. The value co-creation framework builds on the assumption that joint activities can lead to co-creation or co-destruction of value but does not define the meaning of value further. Value, in an LHS sense, is simply put, the best care outcomes for users known as patients, at the lowest possible cost [7].

Co-production and the LHS ideas can potentially provide multiple opportunities to explore and exploit data. This carries the risk of changing the LHS work focus, from having value creation for patients as the primary aim to optimizing other support functions. Efforts to prevent and mitigate risk of losing sight of what is best for the users are therefore essential.

### The LHS to expand engagement and impact

While the LHS literature tends to emphasize technological solutions [9], Osborne *et al*. [3] observe that the roles of technology and of learning have received insufficient attention in early thinking and writing about coproduction. The three assessed LHS models show how new technology, when aptly integrated, can support various types of value co-creation and learning, e.g. by enabling co-evaluation of treatments and of quality improvement efforts. The community-based learning through sharing and building common resources also exemplifies how coproduction can use technology to support learning in an LHS. Furthermore, the three LHSs enable comparison of patient-specific data with corresponding data from all previous patients with similar characteristics, providing individualized information to both patient and clinician on prognosis, treatment options and risks, while building an ever-evolving real-time, real-world evidence base.

The use of measures that matter to the user is central in keeping focus on the aim and considered the core driver of care in an LHS, since the purpose of all efforts is directly or indirectly to generate better health for the ones in need [7, 37, 38].

## Conclusions

The examples of comprehensive LHSs, which exhibit the original IoM LHS characteristics and have been applied in practice, have common features and harness co-production to generate value for individual patients as well as for health systems. These LHS conceptualizations offer a way to expand the role of co-production beyond the original definitions to include ‘all’ stakeholders and increase joint learning and development by integrating supportive technologies into sociotechnical systems. Further research on LHS applications in real-world complex settings is needed to unpack how LHSs are grown through coproduction and other types of value co-creation.
